# Supplementary material for: Transient interactions drive the lateral clustering of cadherin-23 on membrane
Source: Commun Biol. 2023 Mar 18;6:293. doi: 10.1038/s42003-023-04677-6 (PMC10024700; doi:10.1038/s42003-023-04677-6)
Supplement: Supplementary file 2 — Supplementary Information [file 42003_2023_4677_MOESM2_ESM.pdf]

Supplementary Information for

Transient interactions drive the lateral clustering of Cadherin-23 on membrane

Cheerneni S Srinivas[1#], Gayathri S Singaraju[1#], Veerpal Kaur[1#], Sayan Das[1], Sanat K. Ghosh[2], Amin Sagar[1], Anuj Kumar[2,3], Tripta Bhatia[2], and Sabyasachi Rakshit[1,3*]

[*]Correspondence to Sabyasachi Rakshit

Email: srakshit@iisermohali.ac.in

[#] Equal contributions

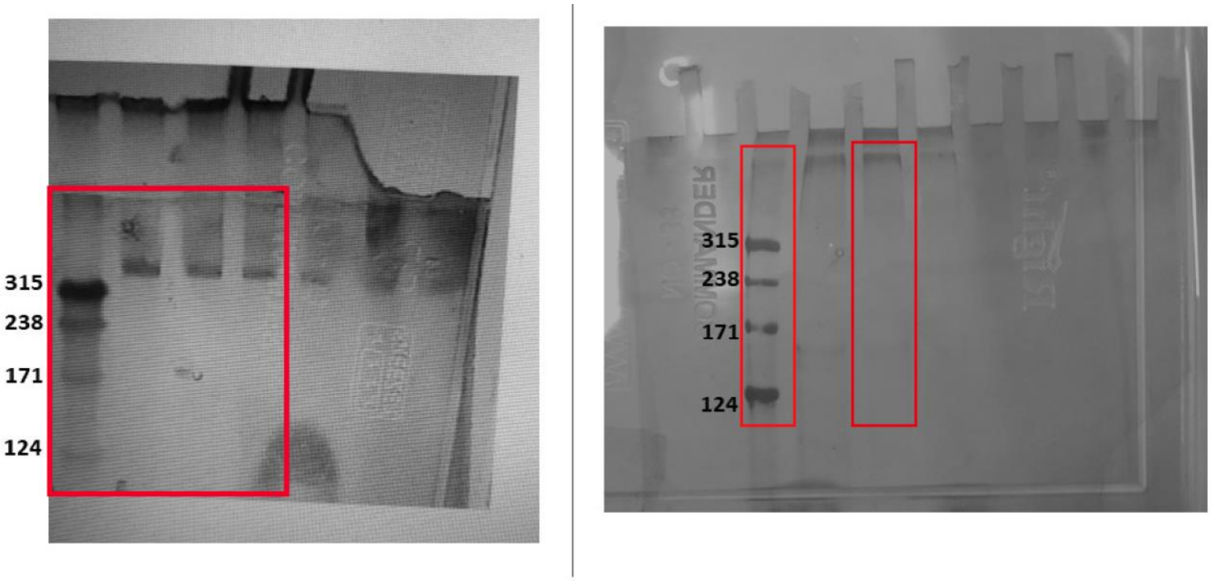

Supplementary Fig. 1 (Supporting to Fig. 1b).

The silver stained uncropped SDS-Page gels showing the monomer (**a**) and trans dimer (**b**) of Cdh23 EC1-27. The rectangles (red) represent the cropped lanes used in the main manuscript.

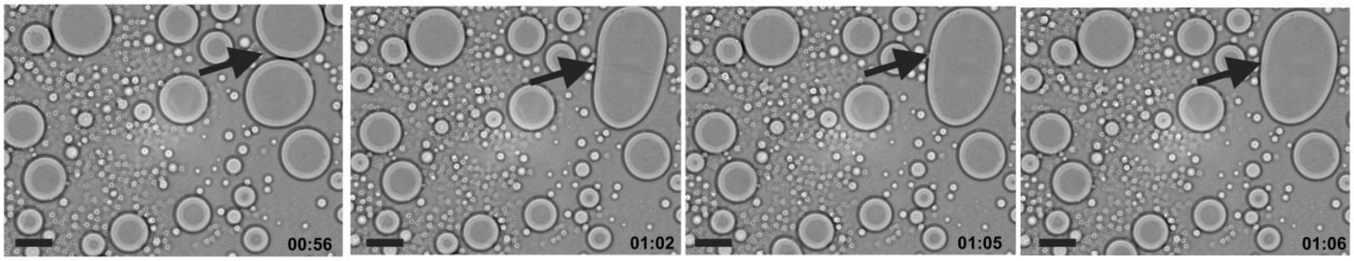

Supplementary Fig. 2: Time-lapse images of droplet fusion (Supporting to Fig. 1c).

The bright-field images with time capture one of the fusion events of liquid droplets of Cdh23 EC1-27. Arrows in black are highlighting the droplets undergoing fusion. Scale bar: 25 µm.

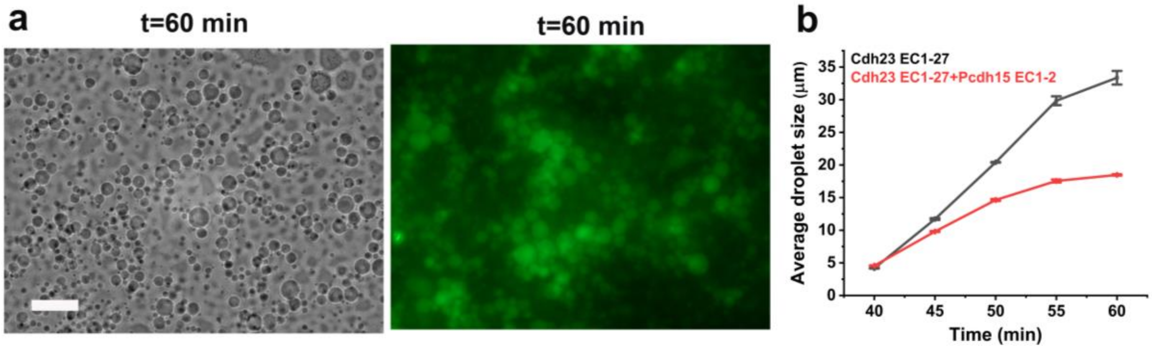

Supplementary Fig. 3: LLPS of Cdh23 EC1-27 in the absence of trans-interactions.

(a) Bright-field and fluorescence images of liquid droplets of Cdh23 EC1-27 induced by exclusive cis-interactions. The trans-interactions were turned off by introducing Pcdh15 EC1-2 in the buffer. The scale bar is 50 µm. **(b)** The comparative growth kinetics of liquid droplets (µm) of Cdh23 EC1-27 in the absence (black) and presence (red) of Pcdh15 EC1-2. Pcdh15 blocks the homophilic trans-binding interface of Cdh23.

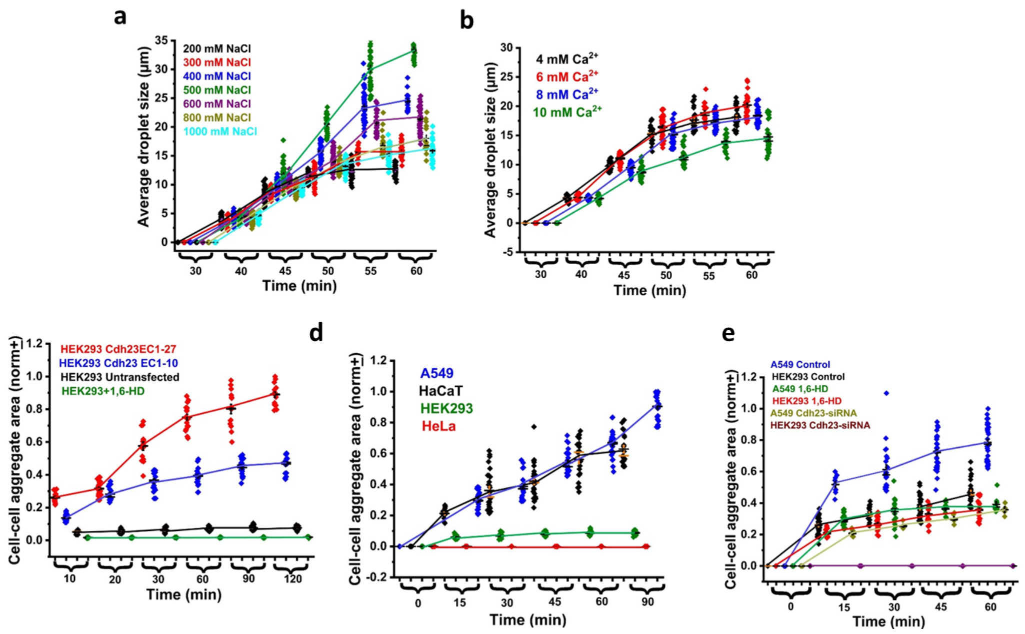

Supplementary Fig. 4 (Supporting to Fig. 1i,j, 3b, d, 4b): Scattered plots containing individual data points for the graphs corresponds to Figures 1i, 1j, 3b, 3d and 4b in the main manuscript.

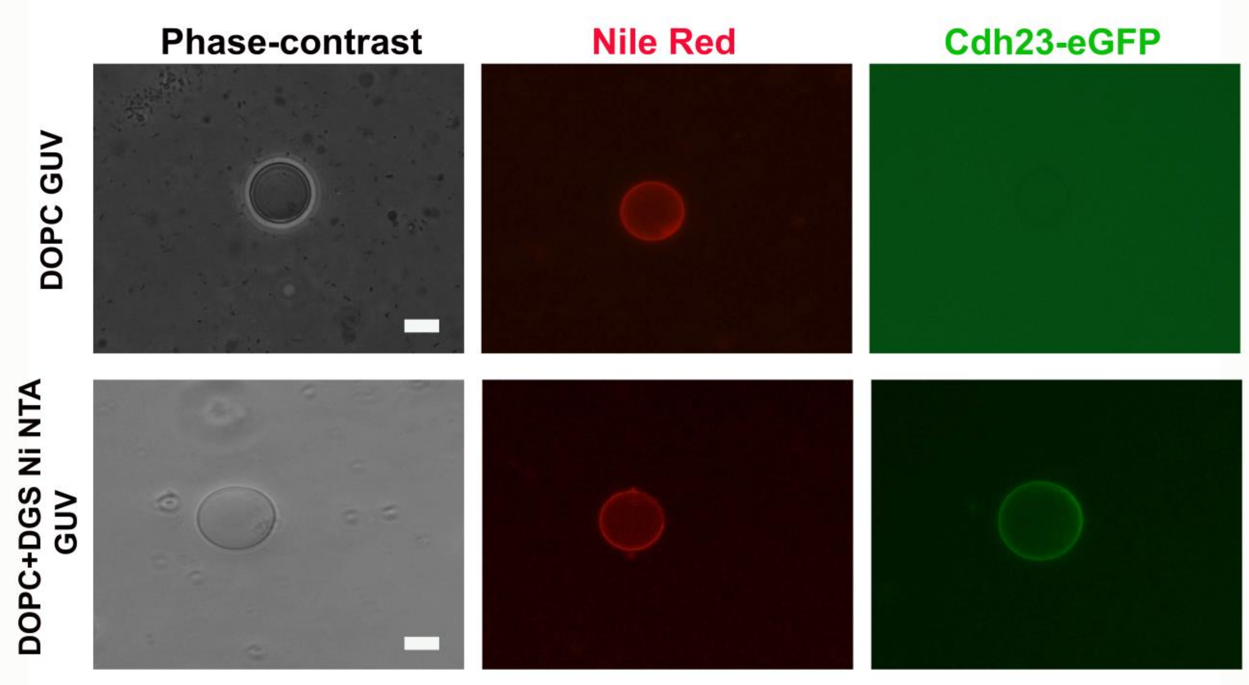

Supplementary Fig. 5: **The specific attachment of Cdh23 to GUV membranes (Supporting to Fig. 2)**.

The representative phase contrast and fluorescence (Nile Red and Cdh23-eGFP) images of DOPC (upper row) and DOPC+DGS Ni NTA GUVs (lower row). Scale bar: 15 μm.

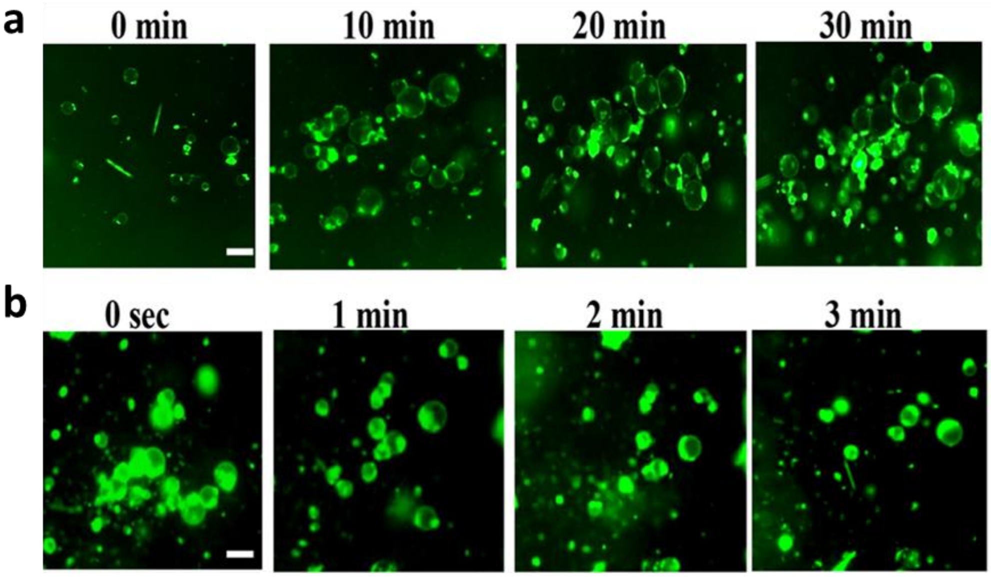

Supplementary Fig. 6:

(**a**) The representative fluorescence images of GUVs specifically attached with Cdh23 undergoing aggregation over time. (**b**) The representative fluorescence images of GUVs specifically attached with Cdh23 detaching from each other in the presence of a calcium chelator, BAPTA. Scale bar: 20 μm.

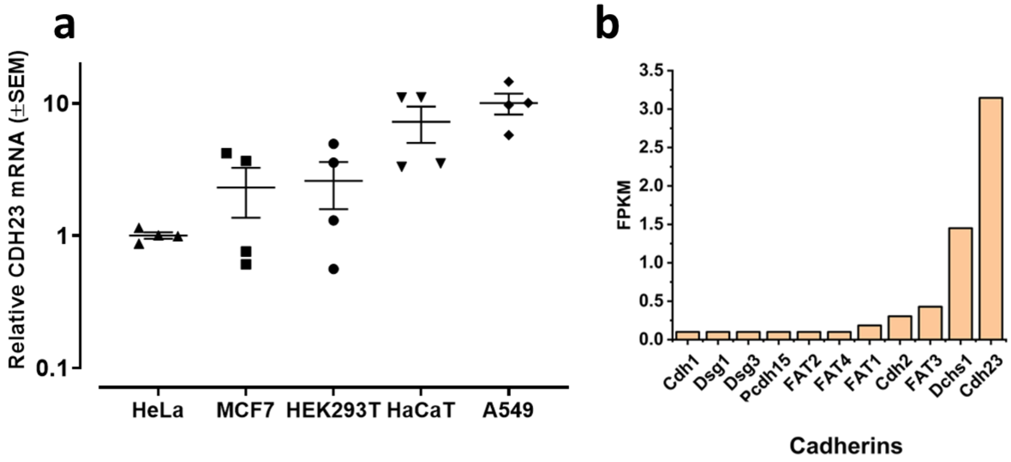

Supplementary Fig. 7: Differential expression of Cdh23 in cancer cell lines and microglia (supporting to Fig. 4).

(**a)** The relative expression of Cdh23 mRNA in different cancer cell lines, namely HeLa, MCF-7, HEK293, HaCaT, and A549, was quantified using qRT-PCR. The highest expression is noticed in A549 and the most negligible expression in HeLa. (**b)** The bar plot displays the mRNA expression of different cadherin proteins in microglia cells. FPKM is Fragments Per Kilobase Million, essentially represents normalized expression values.

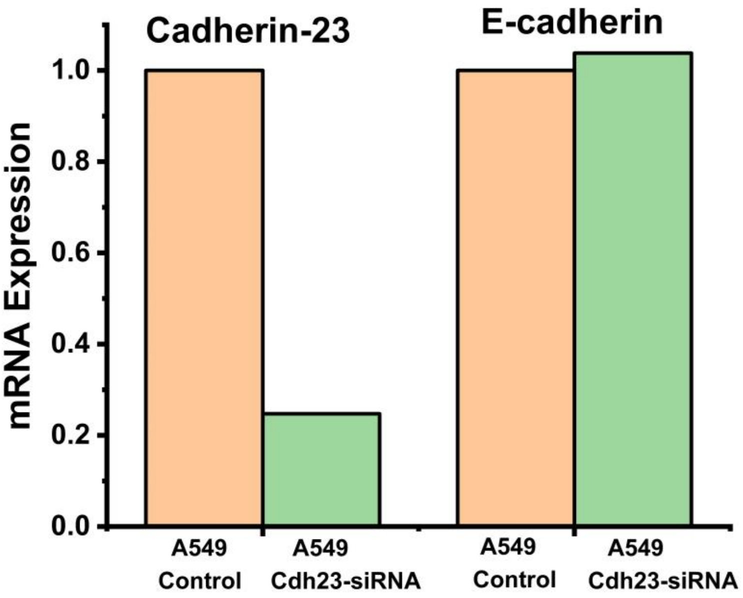

Supplementary Fig. 8 (Supporting to Fig. 4):

Cadherin-23 and E-cadherin mRNA expressions were measured in A549 cells silenced with Cdh23 and control cells using qRT-PCR.

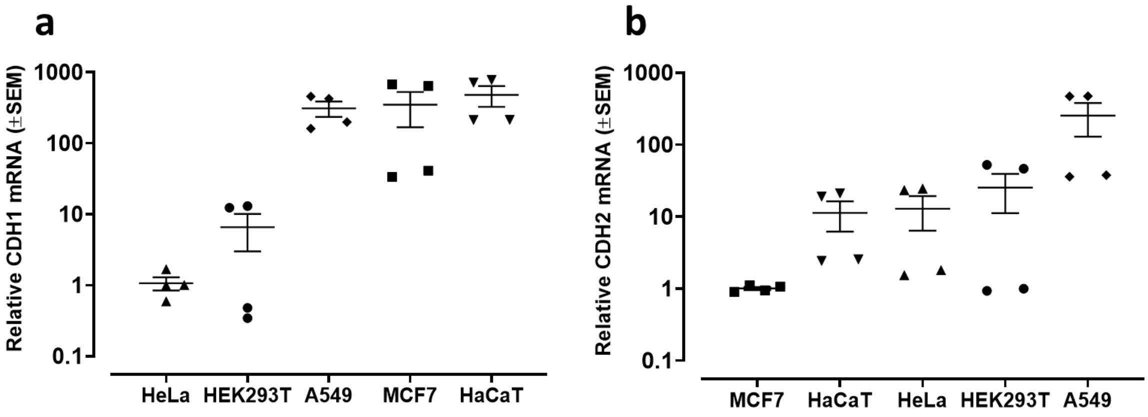

Supplementary Fig. 9:

mRNA level expression of E-cadherin (**a**) and N-cadherin (**b**) was measured in different cell lines using qRT-PCR.

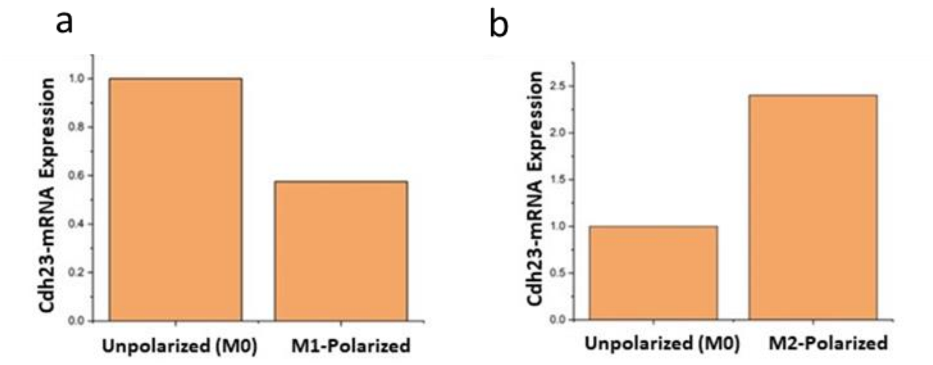

Supplementary Fig. 10:

qRT-PCR estimated that Cdh23 expression is down-regulated in anti-tumorigenic (M1) macrophages (**a**) and up-regulated in tumor-associated (M2) macrophages (**b**). The un-polarized RAW264.7 cells (M0) were polarized to M1 using Lipopolysaccharide and Interferon gamma cytokines, and Interleukin-4 (IL-4) cytokines were used to polarize RAW264.7 cells (M0) to M2.

Supplementary Table 1. Diffusion coefficients measured for the clusters of Cdh23 variants anchored to the pole and periphery of GUVs

Cdh23 variants	Diffusion coefficient ($\mu m^2/s$) at the polar region	Diffusion coefficient ($\mu m^2/s$) at the periphery region
Cdh23 EC1-10	0.61 $\pm$ 0.02	0.52 $\pm$ 0.02
Cdh23 EC1-21	0.13 $\pm$ 0.05	0.12 $\pm$ 0.05
Cdh23 EC1-27	0.23 $\pm$ 0.01	0.24 $\pm$ 0.03

Supplementary Table 2. Diffusion coefficients measured for 1,6-HD and Imidazole treated GUVs labelled with nile red

Experimental condition	*Diffusion coefficient ($\mu m^2/s$)
GUVs labelled with nile red	0.16 ± 0.05
GUVs labelled with nile red + 1,6-HD treated	0.114 ± 0.02
GUVs labelled with nile red + Imidazole treated	0.12 ± 0.04

*The diffusion of Cdh23 is calcium dependent
