# Supplementary material for: Transient interactions drive the lateral clustering of cadherin-23 on membrane
Source: Commun Biol. 2023 Mar 18;6:293. doi: 10.1038/s42003-023-04677-6 (PMC10024700; doi:10.1038/s42003-023-04677-6)
Supplement: Supplementary file 3 — Description of Additional Supplementary Files [file 42003_2023_4677_MOESM3_ESM.pdf]

Description of Additional Supplementary Files

File name: Supplementary Data 1
Description: The source data for the graphs in the paper.

File name: Supplementary Movie 1
Description: Movie showing the fusion of droplets under a fluorescence microscope.

File name: Supplementary Movie 2
Description: Movie showing the fusion of liquid droplets under a bright field.
